# Supplementary material for: Identification of Key Nucleotide Metabolism Genes in Diabetic Retinopathy Based on Bioinformatics Analysis and Experimental Verification
Source: Biology (Basel). 2025 Apr 12;14(4):409. doi: 10.3390/biology14040409 (PMC12024606; doi:10.3390/biology14040409)
Supplement: Supplementary file 1 [file biology-14-00409-s001.zip › Supplementary tables.pdf]

Table S1. The nucleotide metabolism-related genes (NM-RGs).

Table S2. The GO items of differentially expressed NM-RGs (DE-MNRGs).

Table S3. The KEGG pathways of differentially expressed NM-RGs (DE-MNRGs).

Table S4. The subcellular localization of biomarkers.

Table S5. The GSEA results of HMOX1.

Table S6. The GSEA results of TLR4.

Table S7. The GSEA results of ACE.

Table S8. The score between biomarkers and drugs.
