# Supplementary figures and images for: Identification of Key Nucleotide Metabolism Genes in Diabetic Retinopathy Based on Bioinformatics Analysis and Experimental Verification
Source: Biology (Basel). 2025 Apr 12;14(4):409. doi: 10.3390/biology14040409 (PMC12024606; doi:10.3390/biology14040409)

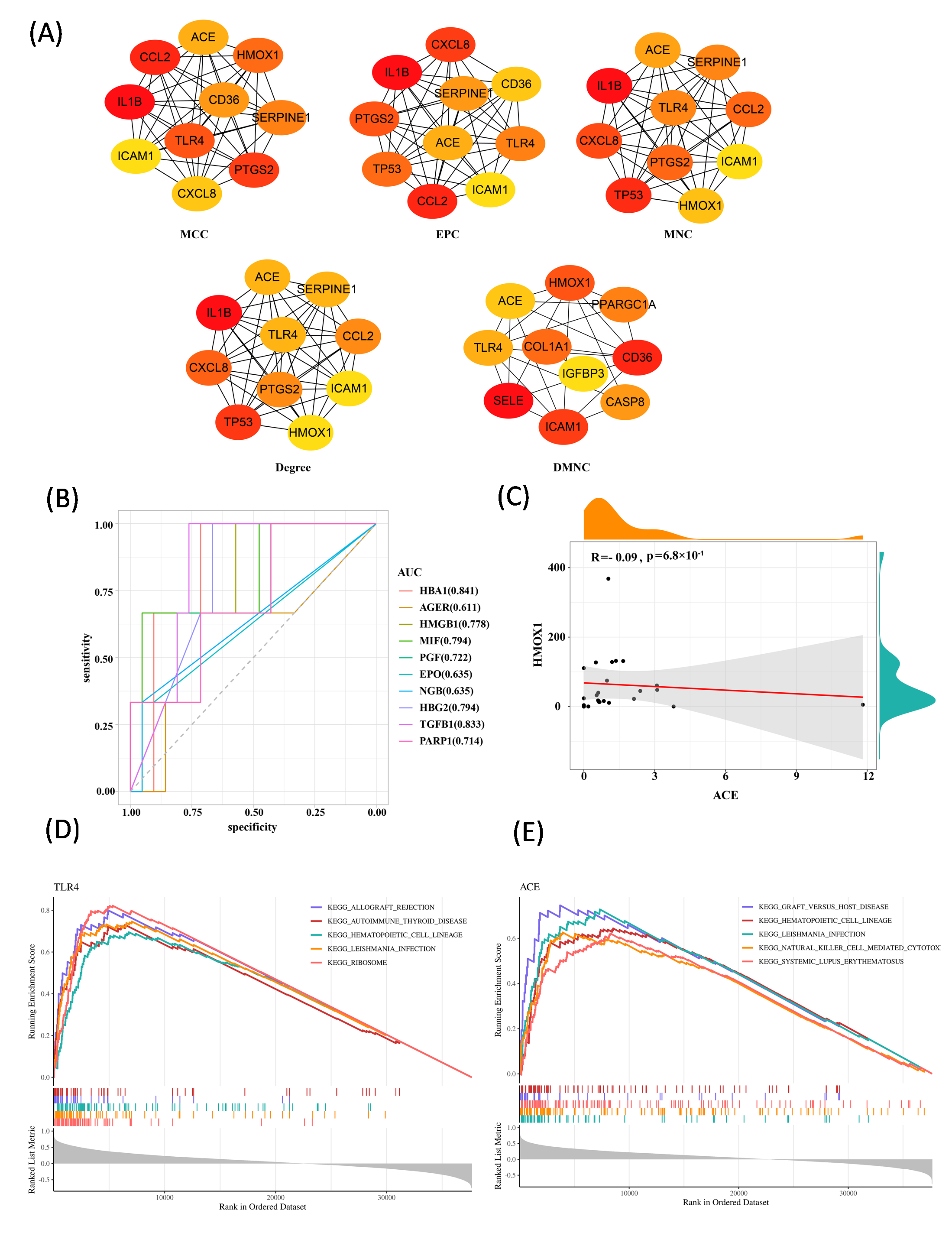

Supplement: Supplementary file 1 [file biology-14-00409-s001.zip › Figure S1.tif]

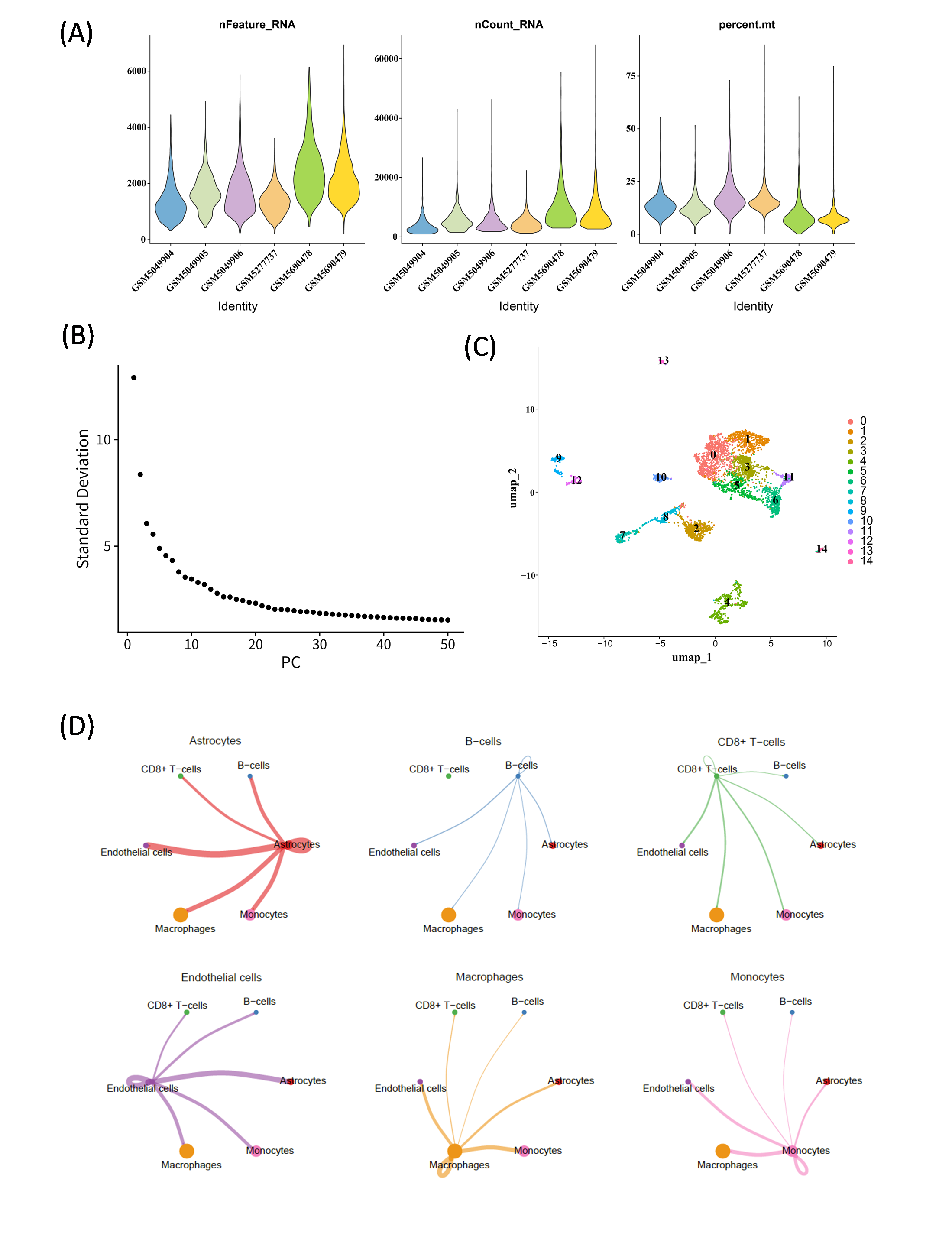

Supplement: Supplementary file 1 [file biology-14-00409-s001.zip › Figure S2.tif]
